# Mental and Physical Health and Intimate Partner Violence against Women: A Review of the Literature

**DOI:** 10.1155/2013/313909

**Published:** 2013-01-23

**Authors:** Gina Dillon, Rafat Hussain, Deborah Loxton, Saifur Rahman

**Affiliations:** ^1^School of Rural Medicine, University of New England, Armidale, NSW 2351, Australia; ^2^Research Centre for Gender, Health and Ageing, University of Newcastle, Callaghan, NSW 2308, Australia; ^3^Faculty of The Professions, University of New England, Armidale, NSW 2351, Australia

## Abstract

Associations between intimate partner violence (IPV) and poor physical and mental health of women have been demonstrated in the international and national literature across numerous studies. This paper presents a review of the literature on this topic. The 75 papers included in this review cover both original research studies and those which undertook secondary analyses of primary data sources. The reviewed research papers published from 2006 to 2012 include quantitative and qualitative studies from Western and developing countries. The results show that while there is variation in prevalence of IPV across various cultural settings, IPV was associated with a range of mental health issues including depression, PTSD, anxiety, self-harm, and sleep disorders. In most studies, these effects were observed using validated measurement tools. IPV was also found to be associated with poor physical health including poor functional health, somatic disorders, chronic disorders and chronic pain, gynaecological problems, and increased risk of STIs. An increased risk of HIV was reported to be associated with a history of sexual abuse and violence. The implications of the study findings in relation to methodological issues, clinical significance, and future research direction are discussed.

## 1. Introduction

Intimate partner violence (IPV) is an entrenched public health and social problem across both developed and developing nations. The World Health Organisation in its 2010 report defines IPV as “behaviour within an intimate relationship that causes physical, sexual or psychological harm, including acts of physical aggression, sexual coercion, psychological abuse and controlling behaviours” [1, page 11]. This definition covers violence by both current and former spouses and intimate partners. There is a growing recognition and understanding of the potential health consequences of IPV both in relation to acute and chronic health impacts beyond the physical trauma cases seen in emergency departments of acute care hospitals and primary care settings. In the past two decades, a growing body of literature has focused on associations between IPV and physical and mental health across a wide range of disciplines. This is reflected in the increasing volume of research articles that deal with psychological consequences and correlates of IPV, including PTSD and other related psychological conditions. 

Since the adoption of the 1993 United Nations General Assembly resolution* Convention on the Elimination of All Forms of Discrimination against Women (CEDAW)* [2], acceptance of IPV as a cross-cultural human rights issue affecting women across the globe has led to international agencies beginning to fund research studies [3–7]. There is now a much broader recognition of the public health implications of IPV, taking it from a personal and family issue related to the legal and justice system to an issue that needs to be acknowledged and addressed at a societal level. Further, increasing evidence suggests that the impact of IPV is not exclusively concurrent with the experience of abuse and may last long after the violence has ceased.

The sheer volume of literature in the IPV area, even when confined to health issues, can create confusion around identifying the most likely correlates and consequences of IPV. This is further complicated by the difficulties researchers face when attempting to assess the health of this population. Studies are frequently confined to drawing samples of convenience (from shelters, clinics, etc.) and where community samples are drawn there are concerns over underreporting—all of which limit generalisability. In addition, the varying ways in which IPV is assessed leads to inconsistency in outcomes across studies. The purpose of this review paper is to provide an overview of recent research literature that has examined IPV and health. The review synthesizes literature from a broad range of studies to map patterns and trends of health consequences and correlates of IPV. 

## 2. Selection Methods

**Figure 1:**
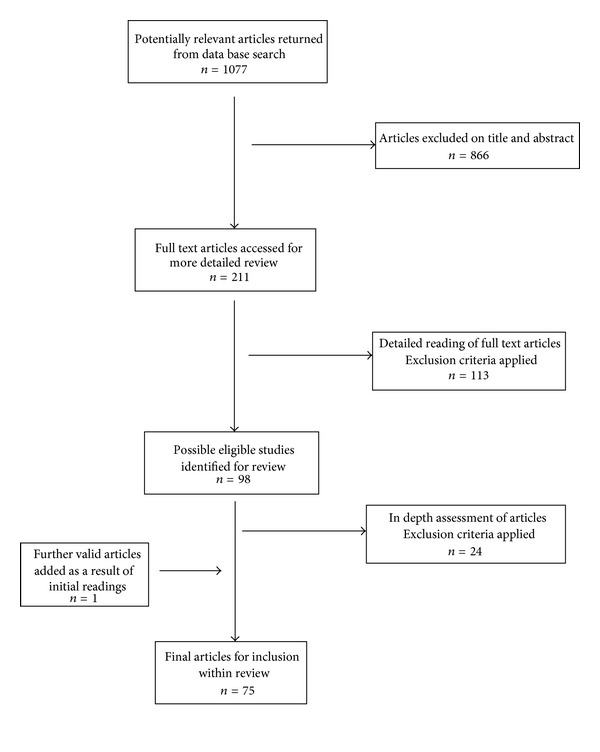
Literature review selection process.

Literature searches of three major online databases (Scopus, SAGE premier, and ProQuest) were undertaken, covering literature published during the time period of January 2006 to June 2012. This time period allowed capture of the most recent papers in the field whilst maintaining the body of reviewed literature to a manageable size. To ensure that papers using a slightly different terminology for IPV were not inadvertently excluded, the search terms used included both “domestic violence” and “intimate partner violence.” As this paper is concerned with the link between violence and health, the terms “physical health” and “mental health” were used in combination with domestic violence and/or intimate partner violence. Boolean operators “AND,” “OR” were included to cover all possible combinations of these search terms. Searches were conducted within the title, abstract, and keyword lists of each database. The process of reviewing articles is outlined in Figure 1.

Articles were firstly assessed on the basis of title and abstract in order to ascertain their relevance for this review. Following this, full-text copies of studies for possible inclusion in the review, were accessed in order to conduct a more thorough evaluation of their relevance.

In determining relevant studies for this review, the following inclusion criteria were used.

- The article reported on an original study, either from primary research or original secondary data analysis.
- Physical and/or mental health consequences or correlates of intimate partner violence were the main foci of the research.
- The research focused on intimate partner violence against women, experienced at any point in adulthood, except during the antenatal or immediate postnatal period.
- The violence defined within the study was restricted to violence against women from a current or previous intimate partner.
- The full text of the research article had to be available in English.
- The article had been published in a peer reviewed journal between January 2006 and June 2012.

Studies that reported on health effects of intimate partner violence for both men and women were included within this review only if the results were stratified by gender, so that the health implications of partner violence on women could be clearly identified. Articles focused on clinical samples were excluded where the specific clinical issues might have compromised generalisability of the results, including samples where IPV occurred during pregnancy or in the immediate postnatal period, studies that focused exclusively on women with specific health condition (e.g., HIV-positive women) and women with specific exposure to additional traumatic events (e.g., military veterans). Studies specifically involving adolescents and high school students were also excluded, since abuse in adolescence was beyond the scope of this review, as were articles relating to health economics.

As illustrated in Figure 1, initial search of the various databases using specified key terms, yielded 1077 articles. These articles were reviewed on the basis of their titles and abstracts using the inclusion and exclusion criteria specified above. This phase of the review removed 866 articles, leaving 211 articles for further consideration. Full-text articles were accessed for each of these studies in order to carry out a more detailed review of their relevance. After application of the specified inclusion and exclusion criteria, the number of articles for review was further reduced to 98 studies. Each of these studies was read and analysed to extract and summarise relevant findings. In this process, an additional 24 papers were removed from the review and one paper was added. Upon completion of the review selection process, there were 75 studies which form the basis for the findings presented in this review. 

## 3. Findings

*Setting of Reviewed Studies and IPV Prevalence*

The study settings for reviewed articles are summarised in Table 1. Just over 30% of the studies utilized a population-based sample and about 19% of the studies collected data from domestic violence shelters. Studies based in medical settings were represented by primary healthcare (13.3%), mental health settings (6.7%), emergency departments (5.3%), and health maintenance organizations (2.7%).

The lifetime prevalence of intimate partner violence reported in the studies varied widely, as indicated in Table 1. In population studies, prevalence ranged from 0.98% [8] to 70.9% [9], whereas in community samples the range was from 11.4% [10] to 44% [11]. The highest reported incidence (78.8%) was from a sample of undergraduate university students [12] reporting on dating violence. Studies that recruited women from domestic violence shelters, or crisis centres for women who had experienced IPV, were reported as a prevalence of 100% as were studies that recruited women who were pursuing protection orders against abusive partners through the court system.

Of the 75 reviewed papers, 55 reported on studies conducted in developed countries and the remaining 20 studies reported on findings from developing countries. The vast majority of studies (*n* = 70) were quantitative in nature, three studies used qualitative methodology and reporting and two studies used a combination of qualitative and quantitative techniques. 

## 4. Health Outcomes

The majority of studies reported findings around the mental health implications of intimate partner violence. Of the 75 studies reviewed, 38 (50%) dealt exclusively with mental health issues, 24 studies (32%) reported on both mental and physical health outcomes, 9 studies (13%) reported on physical health outcomes only, and 4 studies (5%) reported exclusively on sleep problems. In this section, we describe our synthesis of findings sequentially, beginning with mental health outcomes, including depression, PTSD, anxiety, suicidality and self-harm, self-perceived mental health and psychological distress, and impact of intimate partner violence on quality of sleep and sleep disorders. The second section of the review covers findings of intimate partner violence on physical health outcomes including functional health, self-perceived physical health impact, and chronic health conditions. 

### 4.1. Mental Health Outcomes

From the articles included within the review, 66 studies reported on aspects of mental health in relation to intimate partner violence. The mental health conditions reported by each reviewed article are summarized in Table 2.

#### 4.1.1. Depression

Depression was the most commonly researched aspect of mental health in relation to intimate partner violence, being reported on in 42 of the reviewed articles [10, 13–53]. The high relative importance of depression in its impact on health, as a result of IPV, is shown by the burden of disease figures given in the study by Vos et al. [46] who found that 34.7% of the total IPV disease burden was attributable to depression. This is in comparison to 27.3% attributable to anxiety, 10.7% to suicide, and only 0.6% of the burden of disease attributed to physical injuries as a result of IPV [46]. Helfrich et al. [25] reported that the incidence of major depression during the past 12 months was 51.4% from their sample of women's shelter residents. This compared to the national average for the general US female population of just 2.4% reporting depression in the previous 12 months [25].

Of all the studies that investigated the link between depression and IPV history, only one study, conducted in 2008 by Fedovskiy et al. [23], found no significant association between history of IPV and depression. This study of American Latino women (*n* = 105) from a primary care clinical setting found that women endorsing a history of IPV had a higher odds ratio of having a major depressive disorder (OR 1.68) compared to women with no history of IPV that was not statistically significant (*P* = 0.22). Fedovskiy et al. note that their nonsignificant finding is contrary to the findings of previous studies and postulate that the high baseline levels of depressive disorder in their study sample could be a potential confounder that masks any significant effects [23]. 

All the remaining reviewed studies consistently reported significant associations between a history of IPV and depressive symptoms. Several studies indicated that severity or chronicity of violence was associated with more severe depressive symptoms [13, 14, 16, 17, 40, 50]. In contrast to this, the study by Martinez-Torteya et al. [32] indicated that subjective appraisals of the “stressfulness” of an IPV event may have a stronger impact on women's depressive symptoms than more “objective” measures of IPV, such as frequency and severity.

Many studies reported on IPV as a single overarching construct; however, other studies broke down their findings to report on individual categories of violence, most commonly: physical, sexual, and psychological/emotional abuse. Experiencing more than one type of abuse increased the probability of having depressive symptoms as well as the severity of those symptoms [19, 21, 27, 37]. The results reported in the majority of studies indicated that women usually reported more than one type of violence in their history of abuse. Of those studies reporting on depression that did present findings on distinct abuse categories, Pico-Alfonso et al. [37] found psychological IPV to be as detrimental as physical IPV in terms of depressive symptoms in their study sample of Spanish women. Wong et al. [10] found psychological abuse to be the significant predictor of higher levels of IPV-related depression in their study of Chinese women. In this study, it was found that the more frequent the psychological abuse, the higher the level of depression experienced, but this significant result was not found to be present in relation to the frequency of physical abuse [10].

The results reported by Chen et al. [19], in their US-based study of Hispanic women, indicated that women who had experienced sexual abuse from their intimate partner were at far higher odds (OR 42.60, 95% CI: 2.39–758.61) of developing depression than women with either a history of physical (OR 10.28, 95% CI: 1.54–68.77) or psychological abuse (OR 5.83, 95% CI: 2.11–16.16) when compared with nonabused women. The wide fluctuations of the 95% CI for sexual abuse and physical abuse are due to the very small number of respondents in each category, indicating that these results should be viewed with caution. 

 The US-based study by Zlotnick et al. [49] reported on patterns of recovery in mental health status in women with a history of IPV followed across a five-year timeframe. They concluded that women reporting IPV at the commencement of their study were still significantly more likely to experience a greater degree of depressive symptoms and functional impairment with lower self-esteem and life satisfaction at the 5-year followup, compared to women without IPV. They did not find any evidence to suggest that women remaining in an abusive relationship were worse off, in terms of psychosocial difficulties, than women who left those relationships. Thus, they concluded that women who have experienced IPV are at risk of a range of long-term mental health concerns, irrespective of whether or not they stay or leave the abusive relationship [49]. 

#### 4.1.2. Posttraumatic Stress Disorder (PTSD)

Within this review, 14 studies related to the incidence of posttraumatic stress disorder (PTSD) in abused women [15, 18, 21, 23, 25, 27, 30, 32, 33, 36, 37, 54–56]. All studies agreed on the fact that a history of intimate partner violence was positively associated with the increased incidence of PTSD symptoms and PTSD diagnoses. O'Campo et al. [36] estimated that women with a history of IPV were 2.3 times more likely to develop PTSD compared to never-abused women after controlling for race, marital status, and income. Two other studies [23, 33] reported that women with IPV histories had approximately three times the odds of meeting criteria for PTSD as compared to women who did not report a history of IPV.

Within the reviewed studies, the reported prevalence rates of PTSD varied widely. Chandra et al. [18] reported, in their Indian study involving female psychiatric outpatients, that of all the women reporting IPV, 14% met the criteria for PTSD. The rate of PTSD reported from a sample of women from domestic violence shelters in USA was reported as 16.2% by Helfrich et al. [25]. Yet, in a similar sample of abused women from crisis shelters and the general community in USA, sampled by Woods et al. [56], the rate of women who met the criteria for clinical diagnosis of PTSD was much higher at 92.4%. Another US study of women from a health maintenance organisation (HMO) found that 30.9% of women with a history of IPV had symptoms consistent with PTSD, compared with 13.7% of women who did not have a history of IPV [36].

 Similar to the trend for depression, it was reported that women experiencing more severe and more sustained abuse generally exhibited higher levels of PTSD symptoms [18, 27, 56]. Also, the experience of more than one form of abuse led to greater levels of PTSD symptomology [21, 27]. Houry et al. [27] reported that the relative risk of experiencing PTSD symptoms rose with the number of abuse types experienced. Women who had experienced three types of abuse were more than nine times as likely to develop PTSD as a woman who had no history of abuse. A woman experiencing only one type of abuse was just over two times as likely to develop PTSD compared to a nonabused woman [27]. Pico-Alfonso et al. [37] stated that, in their study, the occurrence of PTSD alone was rare, with most women exhibiting comorbidity of PTSD along with depressive symptoms. This appears to be the case in several other PTSD studies as well [15, 18, 23, 36]. This link between PTSD symptoms and depressive symptoms is noted by Fedovskiy et al. [23] who report that women with PTSD were ten times more likely to also have high depression scores (CES-D scores of >15) and they suggest that PTSD and major depressive disorder comorbidity in their study may be a result of symptom overlap, especially the symptoms of anhedonia, sleep disturbance, and concentration difficulties.

#### 4.1.3. Anxiety

Anxiety is often associated with a history of IPV. In this review, anxiety was investigated as a part of sixteen studies [13–15, 20, 22, 25, 28, 31, 37, 39, 40, 43, 46, 50, 51, 53], but it was not the exclusive focus of any of these studies, usually being reported along with other common mental disorders, most often depression. In the study on burden of disease associated with IPV, Vos et al. [46] found that 27.3% of the total IPV burden of disease was attributable to anxiety, making it the second highest contributing factor, with only depression having a higher percentage score. In their US-based study of women from a domestic violence shelter, Helfrich et al. [25] reported that 77% of women from the shelter sample reported anxiety during the previous 12 months, compared to a reported national average of 6.1% for females from a national health survey. 

All sixteen reviewed studies on anxiety reported finding a positive association between a history of intimate partner violence and increased levels of anxiety in women. This relationship existed even after demographic variables such as age, education, and income were taken into account [22, 40, 43]. Pico-Alfonso et al. [37] reported a link between severity of anxiety symptoms and comorbidity with depression, observing that the severity of state anxiety was higher in abused women with depressive symptoms. There was also a dose-response trend apparent, with a greater severity of anxiety symptoms being present in abused women when the abuse experienced was more frequent, more intense, or more severe [13, 40, 50].

#### 4.1.4. Suicide and Self-Harm

From the reviewed articles, six studies reported on suicide attempts [9, 26, 39, 43, 45, 57] and twelve studies on suicidal ideation or thoughts [21, 26, 28, 37, 43, 45, 47, 48, 57–60] in relation to a history of intimate partner violence. All of these studies reported an association between the lifetime experience of abuse and increased suicidal ideation and suicide attempts in women.

Results from the multi-country study by the World Health Organisation on women's health and domestic violence relating to suicide were presented in three separate reports [9, 57, 58]. The paper by Devries et al. [9] dealt specifically with suicide attempts and reported that the experience of IPV was significantly associated with suicide attempts in every one of their thirteen study sites across nine different countries. Ellsberg et al. [57] reported that the pooled analysis, across all of their fifteen sites in ten countries, showed that women who had experienced physical or sexual violence, or both, were three times more likely to have thought about ending their lives and almost four times more likely to have attempted on one or more occasions to have ended their lives, when compared to women who had never experienced partner violence. The reported adjusted odds ratios from a study of South Asian immigrant women resident in the US were even higher, with abused women being seven times more likely to exhibit suicidal ideation than nonabused women from this study sample [26].

In their study of urban Indian women, Vachher and Sharma [45] reported that 22.3% of the study subjects had ever thought of suicide, 12.0% reported suicidal thoughts in the past month, and 3.4% of the women had tried to commit suicide. Suicidal tendencies were considerably more common in women with a history of partner violence, compared to those who had not experienced violence, and these differences were statistically highly significant [45]. The effects of different types of violence in relation to suicidal ideation were reported by Ishida et al. [28] from a population-based study of women from Paraguay. They found that, for abuse in the past 12 months, physical and sexual violence were more important risk factors for suicidal ideation than emotional abuse. For abuse experienced more than 12 months ago, sexual violence had the largest adverse effect, indicating that sexual abuse had a longer lasting negative effect than either of the other two forms of abuse [28]. This is in contrast to the results from Bangladesh, reported by Naved and Akhtar [58], who found that sexual violence by a husband was not associated with suicidal ideation in either rural or urban study sites. They found emotional violence and severe physical violence to be the major determinants of suicidal ideation amongst their sample of Bangladeshi women [58]. The authors also observed a dose-response effect in suicidal ideation in women exposed to a number of forms of violence [58]. Women exposed to no violence, or a single form of violence, had the lowest reporting of suicidal ideation during the previous 4 weeks. An increase in the number of forms of violence, experienced by women in the study, led to an increase in the rate of suicidal ideation reported during the reference period [58]. 

Self-harm was the subject of two reviewed articles [61, 62]. Sansone et al. [61] found that a history of domestic violence was a statistically significant predictor of bodily self-harm in their study of psychiatric inpatients in the USA. A qualitative study of self-harm in victims of intimate partner violence in China [62] revealed that victims considered self-harm to be a method for airing painful emotions caused by abuse, or a last resort to escape by dying when they saw no other option and were no longer able to endure the violence.

#### 4.1.5. Self-Perceived Mental Health and Psychological Distress

The general categories of self-perceived mental health and psychological distress were utilized in nineteen studies. These studies used a range of measurement instruments to report on generalized mental health status and functioning. The Medical Outcomes Study 36-item Short Form Health Survey (SF-36) mental health component score was used in five studies [16, 19, 31, 63, 64] and the shorter SF-12 form in two other studies [65, 66]. Other tools used to measure self-reported mental health status were the WHO developed Self-Reporting Questionnaire (SRQ-20) used in three studies [45, 57, 60], the 12-item General Health Questionnaire (GHQ-12) used in three studies [8, 12, 67] and a development of author's own questions [13, 38] or the use of a range of questions extracted from several survey instruments [49]. A consistent finding of all these studies is that women who had experienced IPV (physical, sexual, or psychological) had lower mental health and social functioning scores than women who had not experienced IPV.

Psychological distress was the reported measure of two studies [68, 69]. Edwards et al. [68] reported on data from a US population-based survey using the Kessler-6 (K6) instrument to measure the degree of serious psychological distress (SPD) experienced in the last thirty days. The risk of SPD was highest among women who reported experiencing both physical and sexual IPV during their lifetime, the prevalence of SPD for this group of women was 15.4%. Among women with no lifetime history of IPV, the prevalence of Serious Psychological Distress was 2.1% [68]. In a Canadian study on IPV in young couples, Fortin et al. [69] used the Psychiatric Symptom Index (PSI) to evaluate psychological distress. It was found for women in the study that a history of psychological violence gave a significant prediction of distress; however, there was no significant prediction of distress in women experiencing physical abuse. It should be noted, however, that the reported prevalence rate of psychological abuse for women in this study (80%) was much higher than the reported rate for physical violence (27%) [69]. 

#### 4.1.6. Sleep Studies

Amongst the reviewed articles, four studies [11, 70–72] focused solely on sleep disturbance as a consequence of intimate partner violence and several others [13, 39, 41, 50] reported on insomnia and other sleep disturbances in the context of broader physical or mental health outcomes. All of the sleep studies support the finding that intimate partner violence has the capacity to impact negatively on both the quality and quantity of sleep in women with experience of IPV. The main mediating pathways between intimate partner violence and poor sleep were reported as depression [11, 70] and PTSD [71].

A qualitative study, reporting on a British focus groups of survivors of partner abuse [72], highlights the dangers of living with a perpetrator of violence and the impacts that this had on their sleep habits. The women stated that being asleep while the perpetrator was awake was seen as extremely risky. For some male perpetrators, their female partner being asleep was sufficient reason for violence, and for others enforcing sleep deprivation was another method of control. Living with a constant anticipation of violence meant that these women felt that they needed to remain vigilant at all times. This could continue long after separation from their violent partner, in some cases 5 or 6 years earlier [72].

As well as disturbed and little sleep, women from the focus group reported problems of aching limbs or teeth grinding, which they related to “sleeping tightly” following the abuse. There were also recurrent bad dreams, including hearing or seeing their ex-partner. Most of the women reported that they had spent considerable periods of time with the quality and quantity of their sleep restricted, and they felt the impact on their health and wellbeing had been significant. The authors concede that it is difficult to make direct links between the women's lack of sleep and physical problems, but the women all felt that their lack of sleep had led to a range of physical health problems. Symptoms reported included being “run down”; aching all over; having migraines and/or headaches, raised blood pressure, chronic fatigue and digestive problems; being more susceptible to other illnesses, such as flu. Sleep deprivation was also reported to dramatically reduce the women's “ability to cope” with the violence they were experiencing [72]. 

### 4.2. Physical Health Outcomes

#### 4.2.1. Functional Physical Health

For the purpose of this review, a study was considered to report functional physical health if it reported on measures of physical functioning and role limitation from the Medical Outcomes Study 36-item Short Form Health Survey (SF-36) [16, 19, 63, 64] or the shorter 12-item version (SF-12) [65, 66, 73] measures, the Patient Health Questionnaire tool (PHQ-15) tool [35] or studies that incorporated their own questions relating to physical health function [25], injury and illness disability [17], and difficulty walking or in performing daily activities [57, 60]. All of the above-mentioned scales are validated tools for measuring physical health. For all but two of the reviewed studies, women with a history of IPV had significantly lower levels of physical functioning than either nonabused women within the same study, or the female national norm value for health function scores. Two studies did not report an association between IPV and functional physical health. In the first, results from a US study by Chen et al. [19] still showed this trend; however, the differences were not statistically significant. The second study, by Helfrich et al. [25] reported that there were no significant differences between their study sample of women from a domestic violence shelter and national norm data on physical symptoms or the effects of those symptoms on function. Detailed data on the physical function of their sample of abused women was not provided in the reviewed article.

Recovery of physical health function after leaving an abusive partner was the subject of one review study [64]. In this Norwegian study, when women were resurveyed 12 months after leaving an abusive partner, it was found that all the SF-36 quality of life domains relating to physical health of the abused women were still significantly lower than the Norwegian female national population of the same age. This suggests that physical functioning levels of abused women are still significantly affected, even after 12 months away from their abusive partners. 

#### 4.2.2. Self-Perceived Physical Health

Self-perceived physical health status, as reported by participants, was included as a health indicator in eight of the reviewed studies. Self-perceived poor health status was significantly associated with intimate partner violence in seven of these studies [17, 26, 33, 38, 60, 66, 67]. As well as physical abuse, nonphysical forms of violence in the form of emotional [60, 67] and psychological [38, 66] abuse were also implicated with lowered levels of perceived physical health. The only study reporting no significant association between the presence of partner violence and lowered levels of reported physical health was Chen et al. [19], as mentioned previously.

#### 4.2.3. Chronic Physical Health Conditions

A history of IPV has been frequently reported to be associated with a range of chronic health conditions. Of the studies included in this review, seventeen studies included consideration of what have been categorized as chronic conditions. These studies and the conditions included are listed in Table 3. 

Chronic pain was reported as being significantly associated with a history of IPV in nine reviewed studies [8, 33, 34, 41, 47, 53, 56, 74, 75] and investigated, but reported as nonsignificant in four additional studies [19, 26, 60, 76]. Loxton et al. [74] reported that pain had one of the highest associations with IPV of all the studied physical symptoms. In a Canadian study of Wuest et al. [75], 35% of the surveyed women reported experiencing high levels of disabling pain even though they had been separated from abusive partners for an average of 20 months. This was significantly higher than the reported Canadian national level of 18% of women reporting debilitating pain [75]. On average, women from the study reported experiencing pain in more than three sites and the authors particularly noted the high prevalence of women (43.2%) reporting swollen and painful joints [75]. Other studies have reported pain as an ongoing health problem for women with a history of IPV, experienced as chronic back ache [8, 56], neck pain [8], stomach cramps [41, 56], and chronic headache [8]. 

The use of pain medication by women with a history of IPV has been reported by several studies [17, 53, 60]. With significantly higher rates of reported chronic pain amongst IPV survivors, it would be expected that the use of analgesics would also be significantly higher amongst these women. However, this was not the case observed in two of the three reviewed studies that dealt specifically with pain medication. Yoshihama et al. [60] found no association between IPV and the use of pain medication, but this study also reported no significant increase in the level of pain reported by the abused women in their Japanese population-based sample that was part of the WHO multi-country study. This finding is contrary to the previously mentioned study of Wuest et al. [75] which found significantly higher pain levels in their community based study sample. Wuest et al. [53], in an allied study on medication use, reported that even though this sample of abused women reported high levels of back pain, headaches, and swollen painful joints, they were less likely to be taking over-the-counter nonsteroidal anti-inflammatory drugs (NSAIDs) and analgesics, and no more likely to be taking opioids than Canadian women in general, even though the incidence of chronic pain was significantly higher. Other chronic health problems that have been associated with the experience of partner violence include cardiovascular and/or circulatory problems (including heart attack, heart disease, hypertension, thrombosis, and stroke) [8, 43, 67, 74, 77]; fatigue, allergies and hearing and sight problems [74]; respiratory problems (including asthma, emphysema, and bronchitis) [42, 74, 77]; bone and muscle conditions (including osteoporosis, arthritis, and other joint problems) [53, 56, 74, 75]; diabetes [67, 74, 77]; low iron [74, 78]; malnutrition and low weight [78] and gastrointestinal conditions [26, 56, 74]. 

Somatoform disorders and psychosomatic complaints were reported in four studies [14, 33, 40, 41]. Scheffer Lingren and Renck report that the women interviewed in their Swedish study suffered various physical symptoms as a result of psychological abuse, including weight loss, weight gain, stomach pain, and pains in “every bone of my body” [41]. Avdibegović and Sinanović [14] also reported a higher incidence of symptoms of somatization among women victims of all categories of IPV in Bosnia and Herzegovina. In a Norwegian study, Nerøien and Schei [33] reported that psychosomatic complaints were more common among women reporting partner violence, with 28% of abused women reporting these complaints compared to 14% of nonabused women. The complaints reported included stomach pain, headache, dizziness, and muscular pain [33]. 

Gynaecological symptoms were reported to be associated with a history of intimate partner violence by women in studies from both developing nations [57, 79] and developed countries [33, 56, 73, 74]. A study by Stephenson et al. [79] found that gynaecological symptoms were significantly related to the reporting of sexual violence, the most common symptom being bleeding after sexual intercourse during times other than menstruation, followed by abnormal vaginal discharge, pain or burning during urination, and pain during intercourse. The incidence of abnormal pap smear results and higher rates of cervical cancer have also been positively associated with a history of IPV [46, 74], as has the occurrence of sexually transmissible infections (other than HIV/AIDS) [43, 46, 80].

Among the reviewed articles, three studies dealt specifically with the association of intimate partner abuse with HIV/AIDS [80–82]. In the population-based Rwandan study conducted by Dude [80], it was found that women who experienced any type of IPV were more likely to report non-HIV STIs, but women who had specifically experienced sexual and emotional abuse were more likely to test positive for HIV compared to nonabused women. Jewkes et al. [81] conducted a two-year study to follow the incidence of new cases of HIV infection among rural South African women. There were a statistically significant higher number of HIV cases reported by women with a history of partner violence (physical or sexual IPV) compared to nonabused women. This was the case both at baseline and also for the newly acquired cases of HIV observed throughout the study period. The HIV risk associated with being in a violent intimate relationship was investigated by Josephs and Abel [82] in a community-based sample of African-American women. They reported that, in their study sample, there was a high correlation between the frequency of physical abuse and sexual coercion. They also showed that violence perpetrated against women by their intimate partners impacted negatively on the women's sexual decision-making and free choice, including the ability to negotiate condom use. This was seen to ultimately increase the risk for HIV [82].

The *WHO multi-country study on women's health and domestic violence against women* is a broad ranging study of the prevalence and health impact of IPV, commissioned by the WHO in 1997. It involved the collection of data from over 24,000 women at fifteen sites in ten countries: Bangladesh, Brazil, Ethiopia, Japan, Namibia, Peru, Samoa, Serbia and Montenegro, Thailand, and the United Republic of Tanzania [4]. Memory loss, problems with concentration, and dizziness were screened for in the World Health Organization questionnaires. Memory loss and problems with concentration were significantly associated with lifetime experiences of partner violence across all study sites in the WHO multi-country study [57] as well as in Japan [60] and Vietnam [47]. Dizziness was also reported as a condition associated with IPV history across several individual studies [33, 56, 57] along with reported neurological complaints [43].

## 5. Discussion

This review has added to current literature by identifying those areas of health most consistently related to experiences of IPV across a wide range of samples, cultures, and ages. By systematically identifying relevant articles and synthesizing the results, it is possible to see that women who have lived with violent partners are more likely than other women to experience a range of psychological and physical symptoms and illnesses, particularly depression, PTSD, anxiety, suicidal ideation, self-harm, insomnia, pain, respiratory conditions, musculoskeletal conditions, cardiovascular disorders, diabetes, and gastrointestinal symptoms. In addition, generally speaking, the more severe and/or the more frequent the experience of IPV, the more severe the symptomatology appears to be, suggesting a dose-response relationship between IPV and depression, PTSD, anxiety, and suicidal ideation.

The inclusion of papers from a range of settings has allowed a broad picture of health outcomes and IPV prevalence to be drawn. Clinical settings, by the nature of the participants, can be expected to report elevated rates of health problems, but the fact that extensive health problems were also consistently reported from a wide range of community and population-based studies attests to the generalisability of results. Studies conducted in health care settings also tended to report higher prevalence rates of IPV than population-based samples. However, many population-based studies had rates of IPV equivalent to or higher than any of the clinical samples, especially those reported from some developing countries within the WHO studies. The scope of papers included within the review could, thus, be seen to reflect a wide range of contexts for IPV, thus portraying a picture of IPV that expands beyond the possible biases of individual study contexts.

Despite the differences in the ways that IPV and health issues were measured and the inclusion of papers that utilised population, shelter and clinical samples from a wide range of cultures, the consistency of findings gives credence to the position that IPV represents a major health and human rights issue. The findings from multi-country studies using standardized questionnaires, as in the two rounds of the WHO multi-country studies as well as standardized modules in the demographic and health surveys (DHS) by many national governments in Asia, Africa, and South America provide similar findings [4, 5]. In almost every country in the world, violence against women is considered a legal crime, yet women may be subject to IPV for many years. There are many reasons why women might remain in violent relationships, including fear, lack of access to legal recourse, lack of resources, cultural norms, and proscription among many others. This review supports the notion that ending violence might lead to increased health and wellbeing, at least for measured scales of vitality and physical function; however, even with this improvement, women with a history of abuse still reported health levels below the national average [64]. However, health deficits related to IPV were also found to last for many years, particularly for psychological conditions such as anxiety, depression, PTSD, and sleep disorders. For other health issues, results across studies were inconsistent regarding improvement after the violence ended. 

Whilst there are methodological limitations in inferring causality from cross-sectional data, the literature on association of IPV and poor health now includes a range of methodological enquiries including longitudinal studies. The presence of a number of longitudinal studies within this review lends a broader perspective to the issue of IPV across the lifespan and helps strengthen confidence in the applicability of findings from cross-sectional studies. Longitudinal studies have indicated similar trends in health outcomes to those found in cross-sectional studies, and this concurrence in findings across methods is striking and provides considerable evidence to support the thesis that a history of IPV precedes poor mental health outcomes that may persist even after the violence has ended. In the current review, eight studies were longitudinal and these allowed for temporal pathways to be shown between IPV and somatic symptoms, poor sleep patterns, HIV infection, and aspects of mental health including depression, anxiety, and PTSD. Less is known about other conditions and this demonstrates a serious gap in the literature. Similarly, results for poor physical health outcomes were consistent across methods. In almost all of the available studies, women's functional health status was poorer and chronic health problems more prevalent compared with other women. These findings point to chronic physical disease mediated through high levels of stress, reduced practice of healthy behaviours [66], and limited agency in deciding on lifestyle choices [82]. The reported findings from the included qualitative research adds a richness and a personal perspective to the understanding of the experiences of women who have had to live with intimate partner violence. This review has some limitations that affect the conclusions that can be drawn. Firstly, there is a known bias towards publication of papers that show significant results rather than those that support the null hypothesis. The current review relied upon published papers, so the degree of contrary evidence, where perhaps results did not show associations between IPV and health is not known. However, the consistency within studies that measured more than one facet of health and the consistency in findings between studies offers strong evidence supporting associations between IPV and poorer health. Causal implications cannot be made from any of the studies examined, although some temporal associations have been found. Again, the review offers a weight of evidence that points to a large population of women who are experiencing poorer health associated with and consequential to IPV. This review utilized three large electronic databases to search for relevant articles for inclusion; however, it is acknowledged that other relevant papers, particularly “grey literature,” may have been missed in this search process. The limiting of the review to English-language-based articles may also have excluded some pertinent papers, especially publications from developing countries which may be more likely to be published in local languages and languages other than English, this is recognized as a limitation to the review process.

The findings of studies included in the present review show that women with a history of IPV experience significantly poorer health including depression, anxiety, PTSD, and reduced measures in both functional and somatic physical health domains. The review also highlighted the need for more methodological clarity in future studies on a number of issues. These include availability of more data on the long-term mental and physical health consequences following IPV through longitudinal studies using standardised definitions and validated scales. There is also a need to better understand the long-term implications of (a) different forms of IPV, (b) the cumulative impact of experiencing multiple types of IPV, and (c) cumulative intensity/severity of IPV. The availability of high-quality cross-cultural qualitative research studies on women's subjective experiences is also of value to allow better triangulation of the data on IPV and adverse health impacts. Future studies also need to focus on the pathways to recovery from abusive experiences and how health services, particularly primary care clinicians, can play a role in this rehabilitative journey. Despite these gaps in current knowledge, it is clear that IPV has serious and long lasting detrimental consequences for women's health and wellbeing. The accumulated findings of the papers within this review underscore the classification of IPV as a public health problem as well as a legal and social issue. 

## Figures and Tables

**Table 1:** Settings and range of IPV prevalence for reviewed studies.

Setting	Studies (*n* = 75)		Reported lifetime prevalence of IPV
	Number	%	Range (%)
Population studies	23	30.6	0.98–70.9
Domestic violence shelter^1^	14	18.7	100
Community sample	13	17.3	11.4–44
Primary healthcare	10	13.3	21.3–39
Mental health setting^2^	5	6.7	46.7–75.9
Emergency department	4	5.3	33.3–54
Health maintenance organisation (HMO)	2	2.7	34–46.4
Education setting	2	2.7	23–78.8
Court system	2	2.7	100

^1^Includes three comparative studies that recruited a sample of abused women from domestic violence shelters and a sample of nonabused women from the general community.

^2^One study from each of the following settings: psychiatric inpatient, psychiatric outpatient, substance abuse clinic, university-based research clinic for mental health problems following IPV, “psychiatric patients” not specified whether inpatient or outpatient.

**Table 2:** Reported mental health conditions following IPV (*✓*: statistically significant; ns: observed, but not statistically significant).

First author Year Reference number	Depression	PTSD	Anxiety	^ 1^Suicide/self-harm	Sleep disorders	^ 2^Poor self- perceived mental health
Alsaker, 2006 [63]						*✓*
Alsaker, 2008 [64]						*✓*
Ansara, 2011 [13]	*✓*		*✓*		*✓*	*✓*
Avdibegović, 2006 [14]	*✓*		*✓*			
Ayub, 2009 [51]	*✓*		*✓*			
Beck, 2011 [54]		*✓*				
Becker, 2010 [55]		*✓*				
Blasco-Ros, 2010 [15]	*✓*	*✓*	*✓*			
Bonomi, 2006 [16]	*✓*					*✓*
Carbone-López, 2006 [17]	*✓*					
Chandra, 2009 [18]	*✓*	*✓*				
Chen, 2009 [19]	*✓*					*✓*
Devries, 2011 [9]				*✓*		
Edwards, 2009 [68]						*✓*
Ehrensaft, 2006 [20]	*✓*		*✓*			
Ellsberg, 2008 [57]				*✓*		*✓*
Escribà-Agüir, 2010 [12]						*✓*
Eshelman, 2012 [21]	*✓*	*✓*		*✓*		
Fadardi, 2009 [22]	*✓*		*✓*			
Fedovskiy, 2008 [23]	ns	*✓*				
Fortin, 2012 [69]						*✓*
Hamdan-Mansour, 2012 [24]	*✓*					
Helfrich, 2008 [25]	*✓*	*✓*	*✓*			
Himelfarb Hurwitz, 2006 [26]	*✓*			*✓*		
Houry, 2006 [27]	*✓*	*✓*				
Ishida, 2010 [28]	*✓*		*✓*	*✓*		
Kim, 2011 [29]	*✓*					
Logan, 2007 [30]	*✓*	*✓*				
Lowe, 2007 [72]					*✓*	
Loxton, 2006 [31]	*✓*		*✓*			*✓*
Ludermir, 2008 [50]	*✓*		*✓*		*✓*	
Martinez-Torteya, 2009 [32]	*✓*	*✓*				
Naved, 2008 [58]				*✓*		
Nerøien, 2008 [33]	*✓*	*✓*				
Nicolaidis, 2008 [34]	*✓*					
Nicolaidis, 2009 [35]	*✓*					
Nur, 2012 [67]						*✓*
O'Campo, 2006 [36]	*✓*	*✓*				
Pico-Alfonso, 2006 [37]	*✓*	*✓*	*✓*	*✓*		
Rauer, 2010 [11]					*✓*	
Renner, 2009 [59]				*✓*		
Roche, 2007 [38]	*✓*					*✓*
Sansone, 2007 [61]				*✓*		
Sato-Dilorenzo, 2007 [39]	*✓*		*✓*	*✓*		
Savas, 2011 [40]	*✓*		*✓*			
Scheffer Lindgren, 2008 [41]	*✓*				*✓*	
Schei, 2006 [42]	*✓*					
Schneider, 2009 [43]	*✓*		*✓*	*✓*		
Stene, 2010 [52]	*✓*					
Straus, 2009 [65]						*✓*
Theran, 2006 [44]	*✓*					
Tomasulo, 2007 [66]						*✓*
Vachher, 2010 [45]	*✓*			*✓*		*✓*
Vives-Cases, 2011 [8]						*✓*
Vos, 2006 [46]	*✓*		*✓*	*✓*		
Vung, 2009 [47]	*✓*			*✓*		
Walker, 2011 [70]					*✓*	
Wong, 2011 [10]	*✓*					
Wong, 2011 [62]				*✓*		
Woods, 2008 [56]		*✓*				
Woods, 2010 [71]					*✓*	
Wuest, 2007 [53]	*✓*		*✓*			
Yang, 2006 [48]	*✓*			*✓*		
Yoshihama, 2009 [60]				*✓*		*✓*
Zlotnick, 2006 [49]	*✓*					*✓*

^1^Suicide/self-harm: includes suicidal ideation and attempts; acts of deliberate self-harm.

^2^Poor self-perceived mental health: low scores on mental health component of SF-36 and SF-12 instruments; reported psychological distress.

**Table 3:** Reported chronic physical conditions following IPV (*✓*: statistically significant; ns: observed, but not statistically significant).

First author Year Reference number	Pain	Fatigue	Allergies	Hearing and eyesight	Respiratory conditions	Bone and muscle	Cardio-vascular	Diabetes	Low iron	Gastro-intestinal
Ackerson, 2008 [78]									*✓*	
Chen, 2009 [19]	ns									
Gass, 2010 [76]	ns		ns		ns	ns	ns	ns		
Gerber, 2008 [73]					*✓*	*✓*		*✓*		
Himelfarb Hurwitz, 2006 [26]	ns									*✓*
Loxton, 2006 [74]	*✓*	*✓*	*✓*	*✓*	*✓*	*✓*	*✓*	*✓*	*✓*	*✓*
Nerøien, 2008 [33]	*✓*									
Nicolaidis, 2008 [34]	*✓*									
Nur, 2012 [67]							*✓*	*✓*		
Ruiz-Pérez, 2007 [77]					*✓*		*✓*	*✓*		
Scheffer Lindgren, 2008 [41]	*✓*									*✓*
Schei, 2006 [42]					*✓*					
Schneider, 2009 [43]							*✓*			
Vives-Cases, 2011 [8]	*✓*						*✓*			
Vung, 2009 [47]	*✓*									
Woods, 2008 [56]	*✓*					*✓*				*✓*
Wuest, 2007 [53]	*✓*					*✓*				
Wuest, 2008 [75]	*✓*					*✓*				
Yoshihama, 2009 [60]	ns									
